# Socioeconomic inequalities in diabetes prevalence: the case of Egypt between 2008 and 2015

**DOI:** 10.1186/s12889-023-16606-7

**Published:** 2023-08-30

**Authors:** Sahar Sidahmed, Siegfried Geyer, Johannes Beller

**Affiliations:** Hannover Medical School, Medical Sociology Unit, Carl-Neuberg-Str. 1, Hannover, 30625 Germany

**Keywords:** Diabetes, Prevalence, Egypt, Social inequalities, DHS, Educational level

## Abstract

**Background:**

There is a steady increase in diabetes prevalence globally and many studies imply that high socioeconomic status (SES) is inversely related to diabetes prevalence. However, there is scarcity in literature from countries like Egypt regarding this topic.

**Methods:**

This study aims to investigate prevalence of diabetes in Egypt between 2008 and 2015, and the effect of SES. Diabetes prevalence -based on self-reports of past diagnosis- was measured using two datasets Egypt DHS 2008 (10,917 participants) and EHIS 2015 (16,485 participants). Logistic regression and odds ratios (ORs) with 95% confidence intervals (CIs) were applied for diabetes controlling for age, gender, educational level, employment status and place of residence. Extend of difference in diabetes prevalence between the two time points was measured by combining the two datasets using the EDHS 2008 as reference.

**Results:**

Diabetes prevalence was higher in 2015 (4.83%) compared to 2008 (3.48%). It was more in women at both time points (4.08% and 5.16% in 2008 and 2015 respectively) compared to men (2.80% and 4.43% in 2008 and 2015 respectively). Older age and living in urban areas were positively related to diabetes prevalence at both time points. Men had a significant higher chance of developing diabetes in 2015 (OR = 1.45, *p*-value = 0.001). Men with higher education had higher chance of developing diabetes (OR = 1.76), in contrast to women (OR = 0.59). Employment decreased the chance of developing diabetes for men (OR = .72), but had minimal effect on women (OR = 1.06).

**Conclusion:**

Diabetes prevalence in Egypt has increased between the years 2008 and 2015 and evident social inequalities were found. Women had more diabetes than men and were more affected with low SES. Unlike women, highly educated men had higher chance of developing diabetes in 2015 compared to 2008. This might be attributed to behavioral and sociocultural factors.

**Supplementary Information:**

The online version contains supplementary material available at 10.1186/s12889-023-16606-7.

## Background

Diabetes is a serious health issue that is progressively affecting more people around the world. The global prevalence of adults with diabetes has risen from 4.7% in 1980 to 8.5% in 2014, especially in low- and middle-income countries (LMIC) [1]. The number of people with diabetes is expected to rise to 417 million by 2030 and to 486 million by 2045 [2]. Diabetes is associated with long-term damage, dysfunction, and failure of various organs, especially eyes, kidneys, nerves, heart, and blood vessels. It is predicted that it will be the 7th leading cause of death in 2030 [3]. A blood sugar reading of 200 mg/dL (11.1 mmol/L) or higher suggests diabetes [4]. There are different risk factors that contribute to the global increase in the prevalence of diabetes, such as population aging, economic development and increasing urbanization. This in turn resulted in physical inactivity and unhealthy diet associated with obesity [2].

The situation in the Eastern Mediterranean Region is very diverse between and within the countries of the region. Malnutrition, undernutrition and food insecurity are common and are associated with high and increasing rates of overweight, obesity and diet-related non-communicable diseases [5]. Diabetes prevalence in the region is expected to increase from 54.8 million people (12.8%) in 2019 to 76.0 million (14.2%) by 2030 and 107.6 million (15.7%) by 2045 [2]. Egypt is the ninth country worldwide and the second in the MENA (Middle East and North Africa) region with the largest number of adults with diabetes (8.9 million) [6]. Results from the survey of noncommunicable diseases (NCD) and their risk factors that was done in Egypt indicated that diabetes prevalence in adults (aged 15–69 years) has increased from 6.0% in 2005, [7] to 15.5% in 2017 [8].

Some of the key risk factors that are associated with high diabetes prevalence in Egypt are obesity and low physical activity. According to the Egypt Demographic and Health Survey (2008), 50% of Egyptian men and 65–80% of Egyptian women are overweight or obese [9]. The regional office of the World Health Organisation (WHO) stated that, the prevalence of insufficient physical activity in adolescents (11–17 years old) is 87.3% (80.6% in boys, 92.9% in girls), and the age-standardized prevalence is 31.0% [5]. Unhealthy dietary habits such as eating meals high in carbohydrates and fats and the growing tendency to consume fast foods are also serious factors that lead to increase in diabetes prevalence [10]. In addition, there are other distinctive risk factors related to Egypt such as increased prevalence of chronic hepatitis C, [11] and increased exposure to environmental risk factors like pesticides [10]. Furthermore, inequalities in access to healthcare services, insufficient training of healthcare professionals and deficient awareness of the symptoms among the general population lead to high numbers of persons with undiagnosed diabetes [2].

In general, health inequalities are defined in respect to unbalanced disease burden or behavioral risk factors that some subgroups of the population are subjected to [12]. The term socioeconomic status (SES) is defined as: “an individual or group’s position within a hierarchical social structure, measured by variables including education, occupation, income, wealth and place of residence” [13]. Although the three main indicators of SES which are: education, occupation and income are usually used reciprocally in research, it is not uncommon to use only one of them [14]. This could be due to small sample size or deficiency of information on other indicators [15, 16]. Even though the gap between high- and low socioeconomic groups is narrowing in certain health indicators, diabetes prevalence is witnessing a widening of this gap [12]. Several studies indicate that people with low socioeconomic status (SES) are at higher risk of developing diabetes. This mainly applies to high-income countries (HIC), whereas in low- and middle-income countries (LMIC) the results are usually different [17]. This could be due to lack of awareness of diabetic symptoms and difficulty in health care access in the lower SES group [18, 19].

### Objectives

Egypt is the ninth country worldwide with the largest number of adults with diabetes, and this number is expected to increase gradually. Understanding socioeconomic characteristics of people who have a higher chance of developing diabetes is an important research area. However, there is deficiency in consistent and reliable data on the influence of SES on diabetes prevalence in Egypt. This study will help organizations and individuals involved or responsible for the delivery of prevention programs, health education and medical services have a good idea about the situation there and be able to look for ways of improvement and development. Researchers will find a scientifically based literature to base their future studies upon.

Using the Demographic and Health Surveys (DHS) Program database, this study aims to examine the prevalence of diabetes in Egypt between 2008 and 2015, taking into account age, gender, and place of residence. SES discrepancies will be examined for by the level of education and employment status.

### Research questions

1. Is there a difference in the prevalence of diabetes in Egypt between 2008 and 2015?
2. Are there any social inequalities regarding the prevalence of diabetes?
3. Is there a difference between 2008 and 2015 in the association of SES with the risk of developing diabetes?

## Methods

### Data

The Demographic and Health Surveys (DHS) Program started in 1984 and provides technical assistance to more than 400 surveys in over 90 countries. It is funded by the U.S. Agency for International Development (USAID) in addition to donors’ contributions from participating countries [20]. DHS surveys intend to collect data on marriage, fertility, family planning, reproductive health, child health, and HIV/AIDS [21]. Information on non-communicable diseases is provided through Woman’s Questionnaire and Man’s Questionnaire [22].

### Egypt Demographic and Health Survey (DHS) 2008

EDHS 2008 collected information from 6578 women and 5430 men aged 15–59 years. The main topics included were knowledge and awareness of avian influenza, HIV/AIDS and hepatitis C; previous history of hypertension, cardiovascular illness, diabetes and liver disease; attitudes and behavior with respect to female circumcision; health care costs; and health insurance coverage. Screening for specific biomarkers was also performed [9].

### Egypt Health Issues Survey (EHIS) 2015

EHIS 2015 had the same objectives as EDHS 2008 and was also implemented in four stages. A total of 27,549 individuals age 15–59 years (9209 women and 7462 men) were interviewed in the 2015 EHIS [23].

### Definition of diabetes cases

The definition of diabetes cases was based on self-reported information. People who responded "Yes" to the question, “has a doctor or other health professional ever told you that you had diabetes?” were classified as having diabetes, and those who responded "No" were classified not to have diabetes. The question did not imply which type of diabetes and no laboratory tests were done to identify the type of diabetes. Since 90% of people with diabetes have type 2 diabetes, [24] participants of this study who reported that they had diabetes were considered having type 2 diabetes.

### Independent variables

Respondents were in the age between 15 and 59 years at the time of interview. They were classified into five age groups with 10 years interval: 15–19 years, 20–29 years, 30–39 years, 40–49 years, and 50–59 years. Based on the highest achieved level of education, there were six categories: no education (no school diploma), incomplete primary education (started primary school but did not finish it), complete primary education (completed 6 years -5 years between 1989 & 2004- of primary education), incomplete secondary education (started secondary school but did not finish it), complete secondary education (completed 6 years in secondary school) and higher education (university diploma or more). Employment status was based on the survey question “Have you done any work in the last seven days even if it was only for a short period of time?”. Participants who answered “yes” were considered “employed”, and participants who answered “no” were considered “not employed”. Place of residence was classified into “rural” and “urban” areas.

### Statistical analyses

To answer the research questions, analyses were performed in three steps. At the first step, descriptive analyses were done for all independent variables and diabetes prevalence was measured. Missing values in the variables “employment” (8 participants (0.07%)) and “diabetes” (4 (0.04%) in 2008) were excluded from the analyses. In addition, participants who responded with “don’t know” in the variable “diabetes,” (48 (0.44%) in 2008 and 12 (0.07%) in 2015) were also excluded from the statistical analysis. Diabetes is a binary variable with two values “yes” and “no.” When prevalence of diabetes by age group was examined, participants in the first three age groups (15–19, 20–29 and 30–39 years) had a low prevalence rate. For the sake of a more precise and uncomplicated statistical analysis, it was agreed upon to merge the first three age groups in one group (15–39 years). At the second step, logistic regression and odds ratios (ORs) with 95% confidence intervals (CIs) was applied for diabetes controlling for age, gender, educational level, employment and place of residence. Adjusted design weights were used in both datasets (2008 and 2015). This was repeated separately for men and women. The first two steps were done for the 2008 and 2015 datasets individually.

At the third step, to measure the extend of difference in the prevalence of diabetes between the two time points, the two datasets were combined using the EDHS 2008 as reference. Logistic regression and odds ratios (ORs) with 95% confidence intervals (CIs) were then applied for diabetes by age, gender, educational level, employment and place of residence.

## Results

### Characteristics of study population

The study population consisted of 10,917 participants (53.47% women) in 2008 and 16,485 participants (55.23% women) in 2015. Almost third of the participants (30.25% in 2008 and 27.15% in 2015) were at the age group 20–29 years. 29.80% of participants in 2008 and 34.43% in 2015 completed secondary school. The percentage of participants without education was 22.05% and 14.75% in 2008 and 2015 respectively. Obvious gender differences were found in the level of education especially in “no education” and “higher” categories at both time points. There was a minimal increase in the percentage of employed participants, from 43.57% in 2008 to 44.42% in 2015. Gender differences were very noticeable, with the majority of men being employed at both time points. In 2008, 57.59% of participants lived in rural areas compared to 50.80% in 2015. No gender differences were observed regarding place of residence. Frequencies for population characteristics including age, gender, educational level, employment status and place of residence are presented in (Table 1).

**Table 1:** Population characteristics stratified by gender and time point

	**2008**		**2015**	
	**Men N (%)**	**Women N (%)**	**Men N (%)**	**Women N (%)**
**N**	5,080 (46.53)	5,837 (53.47)	7,380 (44.77)	9,105 (55.23)
**Age group**				
15–19 years	962 (18.94)	946 (16.21)	1,224 (16.59)	1,371 (15.06)
20–29 years	1,410 (27.76)	1,892 (32.41)	1,849 (25.05)	2,626 (28.84)
30–39 years	1,046 (20.59)	1,301 (22.29)	1,798 (24.36)	2,270 (24.93)
40–49 years	968 (19.06)	1,028 (17.61)	1,366 (18.51)	1,546 (16.98)
50–59 years	694 (13.66)	670 (11.48)	1,143 (15.49)	1,292 (14.19)
**Educational level**				
No education	647 (12.74)	1,760 (30.15)	574 (7.78)	1,857 (20.40)
Incomplete primary	506 (9.96)	464 (7.95)	622 (8.43)	753 (8.27)
Complete primary	281 (5.53)	260 (4.45)	342 (4.63)	354 (3.89)
Incomplete secondary	1,063 (20.93)	968 (16.58)	1,725 (23.37)	1,970 (21.64)
Complete secondary	1,658 (32.64)	1,595 (27.32)	2,767 (37.49)	2,909 (31.95)
Higher	925 (18.21)	790 (13.53)	1,350 (18.29)	1,262 (13.86)
**Employment**				
Not employed	1,211 (23.84)	4,950 (84.80)	1,368 (18.54)	7,794 (85.60)
Employed	3,869 (76.16)	887 (15.20)	6,012 (81.46)	1,311 (14.40)
**Residence**				
Rural	2,918 (57.44)	3,386 (57.70)	3,657 (49.55)	4,718 (51.82)
Urban	2,162 (42.56)	2,469 (42.30)	3,723 (50.45)	4,387 (48.18)

### Diabetes prevalence

In 2008, 3.48% of participants had diabetes in comparison to 4.83% in 2015. Diabetes prevalence was higher in women at both time points, and was consistently positively related to age at both time points. Prevalence of diabetes within women was 4.08% and 5.16% in 2008 and 2015 respectively. On the other hand, 2.80% and 4.43% of men had diabetes in 2008 and 2015 respectively.

### Social inequalities in diabetes prevalence

There was no specific pattern in the prevalence of diabetes for both men and women regarding their educational level in 2008. However, highly educated women had obvious lower diabetes prevalence compared to women without education at both time points (8.08% in women with no education compared to 2.69% in highly educated women in 2015). Highly educated men had somewhat lower diabetes prevalence compared to men without education in 2008, but this was not observed in 2015. Difference in diabetes prevalence was not very distinct between employed and not employed participants in 2008. However, diabetes was more prevalent in employed participants in 2015 especially women (4.80% in not employed compared to 7.32% in employed women). Participants living in urban areas had higher diabetes at both time points, yet it was more obvious in 2015 and in women (3.45% in rural and 7% in urban areas) (Table 2).

**Table 2:** Diabetes prevalence in 2008 and 2015 stratified by age, education, employment and place of residence

	**Diabetes**			
	2008		2015	
	Men N (%)	Women N (%)	Men N (%)	Women N (%)
**N**	142 (2.80)	238 (4.08)	327 (4.43)	470 (5.16)
**Age group**				
15–39 years	21 (0.61)	33 (0.80)	56 (1.15)	61 (0.97)
40–49 years	52 (5.37)	75 (7.30)	89 (6.52)	138 (8.93)
50–59 years	69 (9.94)	130 (19.40)	182 (15.92)	271 (20.98)
**Educational level**				
No education	24 (3.71)	111 (6.31)	29 (5.05)	150 (8.08)
Incomplete primary	19 (3.90)	36 (7.76)	29 (4.66)	76 (10.09)
Complete primary	13 (4.62)	20 (7.69)	15 (4.39)	34 (9.60)
Incomplete secondary	25 (2.41)	22 (2.27)	37 (2.14)	52 (2.64)
Complete secondary	34 (2.05)	35 (2.19)	147 (3.27)	124 (4.26)
Higher	27 (2.76)	14 (1.77)	70 (5.19)	34 (2.69)
**Employment**				
Not employed	27 (2.23)	201 (4.06)	42 (3.07)	374 (4.80)
Employed	115 (2.97)	37 (4.17)	285 (4.74)	96 (7.32)
**Residence**				
Rural	61 (2.09)	97 (2.88)	121 (3.31)	163 (3.45)
Urban	81 (3.75)	141 (5.71)	206 (5.53)	307 (7.00)

After applying logistic regression, significant effects were found in age, gender and place of residence at both time points. In 2008, participants who were 40–49 years old had 10 times the chance of developing diabetes compared to the ones in the (15–39 years) group. Moreover, participants who were 50 years and older had 24 times the chance. This was similarly observed in 2015 where the chance was 8 and 22 times more for the age groups (40–49 years) and (50–59 years) respectively. Women had more chance of developing diabetes at both time points compared to men. This chance was 44% and 21% more in 2008 and 2015 respectively. Urbanization effect was prominent at both time points with 86% and 68% more the chance of developing diabetes in 2008 and 2015 respectively. In 2008, highly educated participants had lower chance of having diabetes, which was not observed in 2015 where there was no specific pattern. This was not statistically significant at both time points. Employed participants had lower chance of developing diabetes at both time points. However, when the analyses were repeated for men and women separately, this effect was only observed in men at both time points. The effect of age and place of residence was more prominent in women (Table 3).

**Table 3:** Odds ratios of diabetes prevalence in 2008 and 2015 stratified by age, education, employment and place of residence for men and women as estimated by means of logistic regression

	**Diabetes**											
	**2008**						**2015**					
	Men *n* = 5080			Women *n* = 5837			Men *n* = 7380			Women *n* = 9105		
	**OR**	**P**	**95% CI**	**OR**	**P**	**95% CI**	**OR**	**P**	**95% CI**	**OR**	**P**	**95% CI**
**Age group**												
15–39 years (Ref.)												
40–49 years	11.52	0.000	6.62–20.03	9.31	0.000	6.00- 14.53	6.43	0.000	4.52- 9.15	9.22	0.000	6.72 -12.67
50–59 years	21.35	0.000	12.56–36.32	26.42	0.000	17.01–41.03	18.45	0.000	13.36–25.47	24.60	0.000	17.97 – 33.68
**Educational level**												
No education (Ref.)												
Incomplete primary	1.30	0.419	.69—2.43	1.11	0.610	.73 – 1.7	1.11	0.711	.65—1.90	1.19	0.266	.87—1.62
Complete primary	1.74	0.127	.85—3.54	.91	0.719	.53 – 1.54	1.27	0.472	.66—2.46	1.18	0.444	.77—1.81
Incomplete secondary	1.89	0.041	1.03–3.50	1.25	0.415	.73–2.12	1.25	0.394	.75- 2.11	1.01	0.952	.71—1.45
Complete secondary	1.27	0.411	.72—2.22	.73	0.181	.46 – 1.16	2.28	0.000	1.45—3.50	1.05	0.751	.78—1.42
Higher	1.35	0.324	.74—2.47	.47	0.026	.25—.92	2.01	0.003	1.26—3.21	.66	0.067	.42—1.03
Employment												
Not employed (Ref.)												
Employed	.66	0.08	.41- 1.05	1.01	0.977	.66- 1.54	.75	0.115	.52- 1.07	1.09	0.535	.82- 1.46
**Residence**												
Rural (Ref.)												
Urban	1.65	0.007	1.15–2.36	2.03	0.000	1.50- 2.74	1.43	0.004	1.12—1.82	1.92	0.000	1.55—2.39

### Difference in the effect of SES on diabetes prevalence between 2008 and 2015

After merging the two datasets with 2008 dataset as reference, participants from 2015 had more chance of developing diabetes than participants from 2008 (OR = 1.25, *P*-value = 0.001). When repeating logistic regression for men and women separately, men had 45% higher risk (*p*-value = 0.001) where women had 13% higher risk of developing diabetes (*p*-value = 0.167) in 2015 compared to 2008. The effect of age and urbanization was more prominent in women than men. Highly educated men had more chance of having diabetes in comparison to men with lower education. On the other hand, highly educated women had relatively lower chance of developing diabetes. Employed men had lower chance of developing diabetes in 2015. However, there was no difference seen in case of employed women (Table 4).

**Table 4:** Odds ratios of time effect on diabetes prevalence stratified by age, education, employment and place of residence for men and women as estimated by means of logistic regression

	**Diabetes**					
	Men *n* = 12,460			Women *n* = 14,942		
	**OR**	**P**	**95% CI**	**OR**	**P**	**95% CI**
**2.wave**	1.45	0.001	1.17—1.78	1.13	0.167	.95—1.34
**Age group**						
15–39 years (Ref.)						
40–49 years	7.75	0.000	5.77 – 10.41	9.25	0.000	7.15—12.00
50–59 years	19.35	0.000	14.69 – 25.49	25.21	0.000	19.54 – 32.54
**Educational level**						
No education (Ref.)						
Incomplete primary	1.18	0.434	.78—1.77	1.16	0.255	.90—1.48
Complete primary	1.44	0.140	.89—2.34	1.06	0.716	.76—1.48
Incomplete secondary	1.43	0.075	.96—2.13	1.06	0.719	.78—1.42
Complete secondary	1.93	0.000	1.38—2.69	.95	0.656	.74—1.21
Higher	1.76	0.002	1.23—2.54	.59	0.005	.41—.85
**Employment**						
Not employed (Ref.)						
Employed	.72	0.027	.54- .96	1.06	0.613	.84- 1.35
**Residence**						
Rural (Ref.)						
Urban	1.49	0.000	1.22—1.82	1.95	0.000	1.64—2.33

## Discussion

### Diabetes prevalence and its association with SES in 2008 and 2015

This study investigated difference in diabetes prevalence and associated socioeconomic inequalities in Egypt between 2008 and 2015. The prevalence of diabetes in Egypt increased from 3.48% in 2008 to 4.83% in 2015, which is in line with reports from the WHO, [25] and the International Diabetes Federation (IDF) [2]. This increase could be due to previously mentioned risk factors such as obesity, physical inactivity, unhealthy diet and smoking. In 2013, Hegazi et al., [10] stated that the prevalence of diabetes in Egypt was about 15.56% in adults between the age of 20 and 79 years. The difference in the percentage could be attributed to the age of participants taking part in this study who were between the age of 15 and 59 years old. Population aging is related to high levels of diabetes, which was presented by the evident age effect in the results of this study that have shown an increase in diabetes prevalence with increasing age for both men and women at both time points.

Although some studies suggest that diabetes is more common in men, results of this study indicate that women have a higher chance of developing diabetes at both time points. Gender differences in diabetes prevalence were acknowledged in the literature. Some studies have found that the chance of developing diabetes was more evident in women, [2, 26] other studies suggest that diabetes is more common in men [27, 28]. In the IDF global atlas of diabetes (2017), diabetes was more prevalent among men (8.9%) at the age of 18–99 years compared to women (8.4%) [2].

Most studies indicate that persons living in urban areas are more at risk of developing diabetes than those living in rural areas. Urbanization is associated with physical inactivity and unhealthy diet. Results of this study coincide with the IDF report where two thirds of people with diabetes live in urban areas [2]. Similar results were found in other countries, such as Algeria, [29] Tunisia, [28] and Oman [30]. Wild et al., [31] argue that even if obesity levels are constant -which is very doubtful-, it is expected that by the year 2030 diabetes prevalence might double due to the effect of urbanization and population aging.

Although the effect of SES on diabetes prevalence was presented in several studies, many researchers have found that women were more influenced by their SES [27, 32, 33]. While there was no clear pattern in the prevalence of diabetes and level of education, it was observed that women with “no education” had higher prevalence of diabetes compared to women with “higher” education. This was observed at both time points with varying degrees. Similar results were found in studies done in Germany, [16] USA, [14] Canada [15] and Tunisia [28]. Men on the other hand, did not show a specific pattern at both time points. Employed participants had higher diabetes prevalence than not employed participants, which was more obvious in women and in 2015. Several hypotheses were brought forward for explaining gender differences in the association of SES with diabetes. Lower SES in women was frequently associated with obesity and increase abdominal waist circumference, [16] which leads to insulin resistance syndrome [34]. In addition, there are several psychological and sociocultural factors that contribute to the risk of developing diabetes [35].

### Difference in risk of developing diabetes between 2008 and 2015 and the effect of SES 

After merging the two datasets, there was an obvious increase in the overall risk of developing diabetes in 2015 compared to 2008 (OR = 1.25, *p*-value = 0.001). This could be due to sociodemographic changes and increased life expectancy, in addition to urbanization which was associated with behavioral and lifestyle changes, unhealthy diet, obesity and physical inactivity.

When examining differences in the effect of SES on diabetes prevalence between the two time points, the main effects of age, gender and place of residence were observed. After repeating logistic regression, gender differences were identified where men had 45% (*p*-value = 0.001) higher risk of developing diabetes in 2015 compared to 2008 while women had 13% (*p*-value = 0.167) higher risk in 2015. Age and urbanization’s effects were more prominent in women than men. The present analysis revealed that highly educated women had lower chance of developing diabetes in 2015 compared to 2008. However, being employed had no effect on women’s chance of developing diabetes. The interesting finding in this study was that highly educated men had a higher chance of developing diabetes in 2015, while employed men had lower chance of developing diabetes. After searching the literature for similar results, a study conducted in Germany by Knopf et al., (1999) observed similar results regarding the prevalence of hypertension and hypercholesterolemia. They stated that it was more observed in men with high SES compared to the ones with low SES but was more common in women with low SES compared to the ones with high SES [36]. The effect of employment was not consistent with education effect in this study. This could be because it was not stated what type of job the participants had. Carlsson et al., found that the type of occupation affects risk of developing diabetes due to associated life style risk factors [37]. Highly educated men in Egypt are more likely to have occupations that are related to office work more than the ones that require physical activity. This in turn leads to a more sedentary lifestyle associated with access to unhealthy type of food, thus increasing chances of obesity. They could also be involved in high-ranking positions that are associated with more stress, which is positively related to diabetes [38]. There is also the possibility of more smoking. In the Global Adult Tobacco Survey (GATS) (2009), they found that the prevalence of smokers in Egypt was 19.4% (37.7% men and 0.5% women) [39].

Results of the present study confirmed previously published literature, and proved that the effect of SES on diabetes prevalence differs between LMIC and HIC. This could be attributed to many factors related to health literacy, [40] lack of awareness with some diabetes symptoms, and difficulty in accessing healthcare services and receiving medical treatment [6]. Other lifestyle factors that could increase diabetes prevalence in Egypt are unhealthy diet and physical inactivity. Although the traditional Egyptian diet is rich in vegetables, fruits, legumes and fish in addition to fair quantities of animal protein, it also comprises a lot of bread, polished rice and fat [10]. While people with high SES consume a lot of fast food, processed meat and dairy products, poor people living in rural areas eat more carbohydrates and fatty diets [10]. Moreover, there is high level of physical inactivity which is more evident in cities and urban areas [5]. Social factors promote that obesity in some areas is not seen as a disease rather as a cosmetic problem that could even be encouraged in some cultures [10]. There are also the previously mentioned distinctive risk factors related to Egypt such as increased prevalence of chronic hepatitis C, [11] and increased exposure to environmental risk factors like pesticides [10].

### Limitations

There are several limitations in this study that need to be noted. Analysis of the present study was based on survey data; therefore, it is expected to have sampling and non-sampling errors. Another limitation is combing two different datasets from two different time points. This could be affected by inaccuracy, inadequacy, and incompleteness of reported data. There is also the possibility of selectivity bias since it is difficult for some people to participate in such surveys because they live in very poor or unregistered households. The high percentage of participants without education could also lead to a greater chance of misunderstanding the questions. Participants' age should be considered as a limitation, since they were between 15 and 59 years old. Although there was a high response rate and the amount of missing data is relatively small, it might have influenced the study findings. In addition, other elements of SES such as participants’ income was not examined since it was not clearly defined in the datasets. As mentioned earlier, this study’s data was based on self-reported diabetes -without doing any lab tests- and it was not implied in the dataset which type of diabetes the participants had. However, it is estimated that almost 90% of people with diabetes have type 2 diabetes [35] and it is difficult to differentiate between the types of diabetes without performing specific laboratory tests. Therefore, participants of this study who reported that they had diabetes were considered having type 2 diabetes. It should be considered that this study’s results were based on data from Egypt, which limits the generalizability of the results to other countries.

## Conclusion

Diabetes prevalence in Egypt has increased between the years 2008 and 2015 and evident social inequalities were found. Age, gender and place of residence were the main influencing factors. Prevalence of diabetes increased with older age and urbanization. Women had higher diabetes prevalence at both time points. Through the time from 2008 to 2015, there was an evident increase in diabetes prevalence associated with obvious gender differences regarding the effect of SES. In contrast to the literature, highly educated men had higher chance of developing diabetes in 2015 compared to 2008. On the contrary, women who were highly educated had lower chance of developing diabetes. This could be attributed to various behavioral and sociocultural factors.

### Supplementary Information

**Additional file 1: Table A. **Diabetes prevalence in 2008 and 2015 stratified by age, education, employment and place of residence.

## Data Availability

All data are fully available without restriction. Researchers can gain access to datasets used for the analyses of this study through registration as DHS data user in this webpage: The DHS Program—login_mainwww.dhsprogram.com/data/dataset_admin/login_main.cfm

## Abbreviations

IDF: International Diabetes Federation
NCDs: Non-communicable diseases
DHS: The Demographic and Health Surveys
SES: Socioeconomic status
LMIC: Low- and middle-income countries
MENA: Middle East and North Africa
EDHS: Egypt DHS
USAID: U.S. Agency for International Development
ORs: Odds ratios
Cis: Confidence intervals
