# Supplementary material for: Socioeconomic inequalities in diabetes prevalence: the case of Egypt between 2008 and 2015
Source: BMC Public Health. 2023 Aug 30;23:1669. doi: 10.1186/s12889-023-16606-7 (PMC10469408; doi:10.1186/s12889-023-16606-7)
Supplement: Supplementary file 1 — Additional file 1: Table A. Diabetes prevalence in 2008 and 2015 stratified by age, education, employment and place of residence. [file 12889_2023_16606_MOESM1_ESM.docx]

**Appendix:** **Table A** **Diabetes prevalence in 2008 and 2015 stratified by age, education, employment and place of residence.**

|  | **Diabetes** | | | | | | | |
| --- | --- | --- | --- | --- | --- | --- | --- | --- |
|  | **2008** | | | | **2015** | | | |
|  | No | | Yes | | No | | Yes | |
|  | **Men N (%)** | **Women N (%)** | **Men N (%)** | **Women N (%)** | **Men N (%)** | **Women N (%)** | **Men N (%)** | **Women N (%)** |
| **N** | 4,938 (97.20) | 5,599 (95.92) | 142 (2.80) | 238 (4.08) | 7,053 (95.57) | 8,635 (94.84) | 327 (4.43) | 470 (5.16) |
| **Age group** |  | | | |  | | | |
| 15-39 years | 3,397 (68.79) | 4,106 (73.33) | 21 (14.79) | 33 (13.87) | 4,815 (68.27) | 6,206 (71.87) | 56 (17.13) | 61 (12.98) |
| 40-49 years | 916 (18.55) | 953 (17.02) | 52 (36.62) | 75 (31.51) | 1,277 (18.11) | 1,408 (16.31) | 89 (27.22) | 138 (29.36) |
| 50-59 years | 625 (12.66) | 540 (9.64) | 69 (48.59) | 130 (54.62) | 961 (13.63) | 1,021 (11.82) | 182 (55.66) | 271 (57.66) |
| **Educational level** |  | | | | | | | |
| No education | 623 (12.62) | 1,649 (29.45) | 24 (16.90) | 111 (46.64) | 545 (7.73) | 1,707 (19.77) | 29 (8.87) | 150 (31.91) |
| Incomplete primary | 487 (9.86) | 428 (7.64) | 19 (13.38) | 36 (15.13) | 593 (8.41) | 677 (7.84) | 29 (8.87) | 76 (16.17) |
| Complete primary | 268 (5.43) | 240 (4.29) | 13 (9.15) | 20 (8.40) | 327 (4.64) | 320 (3.71) | 15 (4.59) | 34 (7.23) |
| Incomplete secondary | 1,038 (21.02) | 946 (16.90) | 25 (17.61) | 22 (9.24) | 1,688 (23.93) | 1,918 (22.21) | 37 (11.31) | 52 (11.06) |
| Complete secondary | 1,624 (32.89) | 1,560 (27.86) | 34 (23.94) | 35 (14.71) | 2,620 (37.15) | 2,785 (32.25) | 147 (44.95) | 124 (26.38) |
| Higher | 898 (18.19) | 776 (13.86) | 27 (19.01) | 14 (5.88) | 1,280 (18.19) | 1,228 (13.85) | 70 (21.41) | 34 (7.23) |
| **Employment** |  | | | | | | | |
| Not employed | 1,184 (23.98) | 4,749 (84.82) | 27 (19.01) | 201 (84.45) | 1,326 (18.80) | 7,420 (85.93) | 42 (12.84) | 374 (79.57) |
| Employed | 3,754 (76.02) | 850 (15.18) | 115 (80.99) | 37 (15.55) | 5,727 (81.20) | 1,215 (14.07) | 285 (87.16) | 96 (20.43) |
| **Residence** |  | | | | | | | |
| Rural | 2,857 (57.86) | 3,271 (58.42) | 61 (42.96) | 97 (40.76) | 3,536 (50.13) | 4,555 (52.75) | 121 (37.00) | 163 (34.68) |
| Urban | 2,081 (42.14) | 2,328 (41.58) | 81 (57.04) | 141 (59.24) | 3,517 (49.87) | 4,080 (47.25) | 206 (63.00) | 307 (65.32) |
